# Supplementary material for: Pyruvate Dehydrogenase Kinase 1 inhibition mediated oxidative phosphorylation enhancement in cartilage promotes osteoarthritis progression
Source: BMC Musculoskelet Disord. 2023 Jul 20;24:597. doi: 10.1186/s12891-023-06585-6 (PMC10357736; doi:10.1186/s12891-023-06585-6)
Supplement: Supplementary file 1 — Additional file 1. [file 12891_2023_6585_MOESM1_ESM.zip › KEGG.Gsea.1653622432464/KEGG_PHENYLALANINE_METABOLISM.html]

Details for gene set KEGG\_PHENYLALANINE\_METABOLISM[GSEA]

|  || Dataset | 55457\_collapsed\_to\_symbols.cls.cls #OA\_versus\_normal.cls.cls #OA\_versus\_normal\_repos |
| Phenotype | cls.cls#OA\_versus\_normal\_repos |
| Upregulated in class | OA |
| GeneSet | KEGG\_PHENYLALANINE\_METABOLISM |
| Enrichment Score (ES) | 0.50498533 |
| Normalized Enrichment Score (NES) | 1.5767394 |
| Nominal p-value | 0.03504673 |
| FDR q-value | 0.04269101 |
| FWER p-Value | 0.621 |
Table: GSEA Results Summary

  

Fig 1: Enrichment plot: KEGG\_PHENYLALANINE\_METABOLISM      
 Profile of the Running ES Score & Positions of GeneSet Members on the Rank Ordered List

  

| SYMBOL | TITLE | RANK IN GENE LIST | RANK METRIC SCORE | RUNNING ES | CORE ENRICHMENT || 1 | GOT1 | glutamic-oxaloacetic transaminase 1 [Source:HGNC Symbol;Acc:HGNC:4432] | 59 | 0.974 | 0.2025 | Yes |
| 2 | MIF | macrophage migration inhibitory factor [Source:HGNC Symbol;Acc:HGNC:7097] | 555 | 0.640 | 0.3000 | Yes |
| 3 | GOT2 | glutamic-oxaloacetic transaminase 2 [Source:HGNC Symbol;Acc:HGNC:4433] | 1308 | 0.457 | 0.3387 | Yes |
| 4 | HPD | 4-hydroxyphenylpyruvate dioxygenase [Source:HGNC Symbol;Acc:HGNC:5147] | 1681 | 0.402 | 0.3952 | Yes |
| 5 | TAT | tyrosine aminotransferase [Source:HGNC Symbol;Acc:HGNC:11573] | 1979 | 0.363 | 0.4493 | Yes |
| 6 | MAOB | monoamine oxidase B [Source:HGNC Symbol;Acc:HGNC:6834] | 2192 | 0.339 | 0.5050 | Yes |
| 7 | PAH | phenylalanine hydroxylase [Source:HGNC Symbol;Acc:HGNC:8582] | 5012 | 0.072 | 0.3014 | No |
| 8 | ALDH3B2 | aldehyde dehydrogenase 3 family member B2 [Source:HGNC Symbol;Acc:HGNC:411] | 5617 | 0.022 | 0.2593 | No |
| 9 | ALDH1A3 | aldehyde dehydrogenase 1 family member A3 [Source:HGNC Symbol;Acc:HGNC:409] | 5831 | 0.007 | 0.2442 | No |
| 10 | ALDH3B1 | aldehyde dehydrogenase 3 family member B1 [Source:HGNC Symbol;Acc:HGNC:410] | 5862 | 0.004 | 0.2428 | No |
| 11 | ALDH3A1 | aldehyde dehydrogenase 3 family member A1 [Source:HGNC Symbol;Acc:HGNC:405] | 6413 | -0.036 | 0.2078 | No |
| 12 | PRDX6 | peroxiredoxin 6 [Source:HGNC Symbol;Acc:HGNC:16753] | 6783 | -0.061 | 0.1922 | No |
| 13 | MAOA | monoamine oxidase A [Source:HGNC Symbol;Acc:HGNC:6833] | 8271 | -0.177 | 0.1143 | No |
| 14 | NAA80 | "N-alpha-acetyltransferase 80, NatH catalytic subunit [Source:HGNC Symbol;Acc:HGNC:30252]" | 8328 | -0.180 | 0.1483 | No |
| 15 | DDC | dopa decarboxylase [Source:HGNC Symbol;Acc:HGNC:2719] | 8605 | -0.202 | 0.1697 | No |
| 16 | AOC3 | amine oxidase copper containing 3 [Source:HGNC Symbol;Acc:HGNC:550] | 9375 | -0.262 | 0.1656 | No |
| 17 | AOC2 | amine oxidase copper containing 2 [Source:HGNC Symbol;Acc:HGNC:549] | 11550 | -0.508 | 0.1048 | No |
Table: GSEA details [plain text format]

  

Fig 2: KEGG\_PHENYLALANINE\_METABOLISM      
 Blue-Pink O' Gram in the Space of the Analyzed GeneSet

  

Fig 3: KEGG\_PHENYLALANINE\_METABOLISM: Random ES distribution      
 Gene set null distribution of ES for **KEGG\_PHENYLALANINE\_METABOLISM**
